# A Two-Year Surveillance of Central Line-Associated Bloodstream Infections in the Trauma ICU of a Tertiary Care Hospital in India

**DOI:** 10.7759/cureus.45325

**Published:** 2023-09-15

**Authors:** Safia Maqbool, Rajni Sharma

**Affiliations:** 1 Medicine, Sawai Man Singh (SMS) Medical College and Hospital, Jaipur, IND; 2 Microbiology, Sawai Man Singh (SMS) Medical College and Hospital, Jaipur, IND

**Keywords:** central line-associated bloodstream infection, hospital-acquired infections, trauma icu, intensive care unit, central venous catheter

## Abstract

Aim

The aim of the study is to identify the risk factors and mortality associated with central line-associated bloodstream infection (CLABSI) and to investigate the incidence and associated etiology in trauma patients admitted to the trauma ICU (TICU) of a tertiary care teaching hospital in Northern India.

Materials and methods

The study was a prospective study conducted in the trauma ICU of a tertiary care teaching hospital in India from November 2020 to October 2022. Adult patients >18 years of age who were on central line for >48 hours were included in the study. The automated blood culture system BacT/ALERT 3D (bioMérieux, Durham, NC) was used for microbial detection from blood samples. We recorded patients' daily progress, and catheter-related data was collected and used as variables. All the data was analyzed using the Statistical Package for Social Sciences (SPSS) version 22.0 (IBM SPSS Statistics, Armonk, NY) to evaluate the risk factors associated with CLABSI.

Result

A total of 516 admissions occurred during the surveillance period, out of which 352 patients fulfilled the inclusion criteria and were enrolled in the study. Out of these 352 patients, a total of 74 patients developed central line-associated bloodstream infection (CLABSI). Thus, the incidence of CLABSI was 16.4 per 1000 central line days and 13.2 per 1000 inpatient days with a 0.8 device utilization ratio (DUR). The most common organisms isolated from these CLABSI cases were *Acinetobacter* species (23%), followed by *Escherichia coli* (16.5%) and *Staphylococcus aureus *(15.6%). The independent healthcare-associated risk factors for CLABSI were longer length of ICU stay and prolonged duration of central venous catheterization. The most common comorbidity associated with CLABSI was diabetes mellitus (20.3%), followed by hypertension (14.8%), and the mortality rate was 41.9%.

Conclusion

The healthcare-associated risk factors such as longer length of ICU stay and prolonged duration of central venous catheterization are the risk factors for developing central line-associated bloodstream infections (BSI).

## Introduction

Central venous catheterization is frequently used for monitoring and managing critically ill patients [1]. As these catheters have access to the bloodstream, they can lead to significant complications, most importantly bloodstream infections (BSI). Central line-associated bloodstream infection (CLABSI) is the most common healthcare-associated infection (HAI) [2,3]. It is one of the leading causes of morbidity and mortality in hospitals across the world [4]. The development of CLABSI is due to several risk factors [5,6]. The prolonged duration of catheterization is a major risk factor, as the longer a patient has a central line in situ, the higher the risk of developing CLABSI [7]. The reason is that bacteria colonize the catheter and lead to the formation of biofilms, making it difficult to treat the infection. Critically ill patients are vulnerable to serious complications due to progressively more invasive procedures, the presence of multiple invasive devices, immunocompromised status, old age, comorbidities, and the higher incidence of antimicrobial resistance [8]. In trauma patients, most often, central line insertions are performed in an emergency, thus increasing the risk of infection [9]. Other risk factors include inadequate bundle care preventive measures, poor patient/nurse ratio, and unqualified staff in infection prevention and control (IPC) practices [8]. Central line-associated bloodstream infections (CLABSIs) are associated with increased mortality from 4% to 37%, in addition to prolonged hospital stays and increased healthcare costs [10,11].

Patients with CLABSI are at higher risk of developing sepsis, organ failure, and other life-threatening complications [12]. Furthermore, CLABSIs can lead to multidrug-resistant infections, which are challenging to treat and increase the risk of poor outcomes [13]. The objective of this study was to identify the risk factors associated with CLABSI in adult trauma patients admitted to the trauma ICU (TICU) and to investigate the incidence and etiology of CLABSI.

## Materials and methods

Aim

The aim of the study is to identify the risk factors and mortality associated with CLABSI and to investigate the associated incidence and etiology in the trauma ICU of a tertiary care teaching hospital in Northern India.

Method

This prospective study was conducted in the department of microbiology and trauma ICU (TICU) of the Sawai Man Singh (SMS) Medical College and Attached Hospitals, Jaipur, Rajasthan, from November 2020 to October 2022 after obtaining ethical approval from the Ethics Committee with institutional review board (IRB) number 281MC/EC/2021. All patients admitted to the TICU who were >18 years old with a central line inserted at our hospital for >48 hours were included in the study. Consent was obtained from each patient. Patients with secondary BSI were excluded. A total of 352 patients were included in the study, as per the sample size calculated using a 95% confidence interval and 5% margin of error.

Data was prospectively collected daily using a standardized format for all patients admitted to the TICU meeting the inclusion criteria. Patients were monitored until discharged or two days following their transfer to a ward in the same hospital. The total number of patient admissions in the TICU was also monitored during the study period. For each patient, the records included demographic data and data on chronic conditions, the length of ICU and hospital stay, requirements for other indwelling devices, previous infections, exposure to antibiotics, and mortality. All patients included in the study were analyzed for various risk factors such as age, surgery/trauma, duration of hospitalization, duration of central line, and comorbidities. These factors were analyzed for their potential role in predicting the risk of developing CLABSI and its attributable mortality.

Criteria for CLABSI

The criteria for CLABSI were if a recognized pathogen was cultured from one or more percutaneous blood cultures 48 hours after vascular catheterization and if the pathogen was unrelated to an infection at another location or if a common commensal such as diphtheroids, *Bacillus* spp., *Propionibacterium* spp., and coagulase-negative staphylococci or micrococci was cultured from two or more blood cultures drawn on separate occasions along with one of the following signs: fever of >38 degrees Celsius or hypotension [14]. On the clinical suspicion of sepsis, blood specimens were drawn from peripheral venipuncture/lumen of central line as per standard protocol and sent to the department of microbiology for culture and sensitivity testing. To exclude the secondary bacteremia samples from other sites such as urine, endotracheal (ET) secretions and pus were also collected, and if no other source was found, then the infection was considered as sepsis due to intravascular catheterization.

Sample processing

THE automated blood culture system BacT/ALERT 3D (bioMérieux, Durham, NC) was used, and samples were incubated at 37 degrees Celsius for up to five days. Positive blood samples were subcultured for identification on blood agar and MacConkey agar and the organisms were identified manually by Gram stain, colony characteristics, and biochemical tests as per standard laboratory protocol [15,16]. The Kirby-Bauer disc diffusion method was used for testing antimicrobials as per the Clinical and Laboratory Standards Institute (CLSI) guidelines (2020) [17].

Calculation of incidence

CLABSI rate was calculated by the total number of reported CLABSI/number of central line days multiplied by 1000, and the device utilization ratio (DUR) was calculated by the number of device days/number of patient days [14].

Statistical analysis

Statistical analysis was performed using percentage, mean, median, standard deviation, and t-test. A p value of <0.05 was considered significant. Statistical data was compiled, tabulated, and examined statistically using the Statistical Package for Social Sciences (SPSS) version 22.0 (IBM SPSS Statistics, Armonk, NY) to obtain valid results.

## Results

A total of 516 admissions occurred from November 2020 to October 2022, out of which 352 patients fulfilled the inclusion criteria and were enrolled in the study accounting for 4523 catheter days and 5615 inpatient days. Out of these 352 patients, a total of 74 patients developed central line-associated bloodstream infection (CLABSI). Thus, the incidence of CLABSI was 16.4 per 1000 central line days and 13.2 per 1000 inpatient days with a 0.8 device utilization ratio.

We observed the predominance of Gram-negative organisms (77%), followed by Gram-positive organisms (22%). We also observed a low prevalence of *Candida* species (0.9%) (Table 1). A total of 109 microorganisms were isolated from these 74 cases of CLABSI as 23 (31.8%) samples showed polymicrobial growth in blood culture. The most common organisms isolated from these CLABSI cases were *Acinetobacter* species (23%), followed by *Escherichia coli* (16.5%) and *Staphylococcus aureus* (15.6%) (Table 2). The highest antimicrobial sensitivity was observed against polymyxin B (100%), followed by tigecycline (93.3%) and minocycline (63.3%), in Gram-negative organisms. In Gram-positive organisms, 100% sensitivity was displayed against vancomycin and teicoplanin. We found that one (5.9%) isolate of *Staphylococcus aureus* was resistant to linezolid and one (14.3%) isolate of the *Enterococcus* species was resistant to teicoplanin. All the 17 (100%) isolates of *Staphylococcus aureus* were resistant to methicillin. Out of the seven isolates of the *Enterococcus* species, two (26.6%) isolates were vancomycin-resistant *Enterococcus* (VRE).

**Table 1:** Nature of microorganisms associated with central line-associated bloodstream infection (CLABSI) The table shows the commonly associated microorganisms with central line-associated bloodstream infections

Nature of microorganism	n (%) (total=109)
Gram-negative organisms	84 (77%)
Gram-positive organisms	24 (22%)
*Candida* species	1 (1%)

**Table 2:** Microorganisms associated with central line-associated bloodstream infection (CLABSI) The table shows the microorganisms associated with central line-associated bloodstream infection MRSA: methicillin-resistant *Staphylococcus aureus*

Microorganisms isolated	n (%) (total=109)
*Acinetobacter* species	25 (23%)
E. coli	18 (16.5%)
*S. aureus* (MRSA)	17 (15.6%)
Klebsiella pneumoniae	13 (12%)
Enterobacter aerogenes	12 (11%)
*Enterococcus* species	7 (6.4%)
Pseudomonas aeruginosa	7 (6.4%)
Burkholderia cepacia	4 (3.7%)
Proteus mirabilis	4 (3.6%)
*Citrobacter* species	1 (0.9%)
*Candida* species	1 (0.9%)

The incidence of central line-associated bloodstream infection was higher in males (82.4%) as compared to females (17.6%). In the trauma ICU, younger age group of 21-30 years (32.4%) commonly developed CLABSI, followed by 31-40 years (23%), with a mean age of 36.2±14.1 years (Table 3).

**Table 3:** Frequency of central line-associated bloodstream infection (CLABSI) in different age groups The table shows that young age group commonly developed central line-associated bloodstream infections in the trauma ICU

Age group of CLABSI patients (years)	n (%) (total=74)
≤20	7 (9.4%)
21-30	24 (32.4%)
31-40	17 (23%)
41-50	9 (12.2%)
51-60	14 (19%)
61-70	3 (4%)

We observed that the healthcare-associated risk factors such as longer length of ICU stay and prolonged duration of central venous catheterization were associated with the increased incidence rate of CLABSI. The highest CLABSI cases were from patients with longer length of ICU stay of >11 days (77%), followed by >5 days to ≤10 days (13.5%), with a mean of 19±18 days (Table 4). The minimum length of ICU stay of these CLABSI patients was three days, and the maximum was 96 days with a median (interquartile range {IQR}) of 15 (9-21) days. As per Table 5, the highest CLABSI cases were from patients with a prolonged duration of central venous catheter (CVC) (>11 days) in 56.8%, followed by >5 days to ≤10 days in 28.4%, with a mean of 18.13±10.02 days. The minimum duration of central venous catheterization in these CLABSI patients was three days, and the maximum duration was 47 days with a median (IQR) of 17 (10-24) days. The independent healthcare-associated risk factors such as longer length of ICU stay and prolonged duration of central venous catheterization were statistically highly significant (p value of <0.05) (Table 6).

**Table 4:** Association of central line-associated bloodstream infection (CLABSI) with the length of ICU stay The table shows that the longer length of ICU stay is a risk factor for central line-associated bloodstream infection

Length of ICU stay (days)	n (%) (total=74)
>2 days to ≤5 days	7 (9.5%)
>5 days to ≤10 days	10 (13.5%)
>11 days	57 (77%)

**Table 5:** Association of central line-associated bloodstream infection (CLABSI) with the duration of central venous catheterization The table shows that the prolonged duration of a central venous catheter is a risk factor for central line-associated bloodstream infection

Duration of central venous catheterization (days)	n (%) (total=74)
>2 days to ≤5 days	11 (14.8%)
>5 days to ≤10 days	21 (28.4%)
>11	42 (56.8%)

**Table 6:** Comparing the risk factors of patients with and without nosocomial infections The table shows that longer length of ICU stay and prolonged duration of a central venous catheter were independent risk factors (p<0.05, by t-test) for central line (CL)-associated bloodstream infections. ***Highly significant TICU: trauma ICU

Variables	Infected	Uninfected	P value
Age	36.89±14.51	40.52±16.7	0.54999
TICU duration	22±19.94	9.26±10.19	0.00000***
CL duration	18.13±9.44	8.83±6.46	0.00000***
Mortality	31 (41.9%)	175 (63%)	0.46478

As per Table 7, 58.1% of patients who were diagnosed with central line-associated bloodstream infection (CLABSI) had no comorbidity, whereas 49.1% of patients had comorbidity. The most common comorbidity associated with CLABSI was diabetes mellitus (20.3%), followed by hypertension (14.8%). Out of 74 patients who were diagnosed with CLABSI, 31 (41.9%) patients scummed to death, and 37 (50%) recovered and were discharged, whereas in patients with no BSI, out of 278 patients, 175 (63%) patients expired, and 99 (35.6%) recovered and were discharged.

**Table 7:** Frequency of comorbidities associated with central line-associated bloodstream infection (CLABSI) The table shows comorbidities associated with central line-associated bloodstream infections CKD, chronic kidney disease; COPD, chronic obstructive pulmonary disease

Comorbidity	n (%) (total=74)
Diabetes mellitus	15 (20.3%)
Hypertension	11 (14.8%)
CKD	1 (1.4%)
COPD	4 (5.4%)
No comorbidity	43 (58.1%)

## Discussion

Central line-associated bloodstream infection rates in ICUs of developing countries are higher than in the developed world. They are associated with increased hospitalization and higher healthcare costs, and in fact, out of all types of hospital-acquired infections, they are associated with higher morbidity and mortality. Therefore, the aim of this study was to identify the risk factors associated with CLABSI and to investigate the incidence and associated etiology in the trauma ICU in a tertiary care teaching hospital in Northern India.

A total of 352 patients fulfilled the inclusion criteria and were enrolled in the study. Out of these 352 patients, 74 patients developed central line-associated bloodstream infection (CLABSI). Thus, the incidence of central line-associated bloodstream infection (CLABSI) was 16.4 per 1000 central line days and 13.2 per 1000 inpatient days with a 0.8 device utilization ratio. Comparable results were reported by Singh et al. [18], Chopdekar et al. [19], and Ben Jaballah et al. [20], who reported incidences of 16.6, 27.065, and 15.3 per 1000 catheter days. We had a high incidence of central line-associated bloodstream infection in the trauma ICU as compared to the other studies reported by authors such as Parameswaran et al., who reported a CLABSI incidence of 8.6/1000 catheter days [21]. In our study, the reason for the high incidence might be that trauma patients are on multiple invasive devices, which act as portals of colonization and invasion for pathogens. Moreover, most of the head-injured patients are on ventilators, predisposing them to secondary bacteremia. In trauma patients, most of the time, central line insertions are carried out in emergencies, and strict aseptic precautions are often neglected. Moreover, hemodynamic instability and the need for repeated blood transfusions necessitate the insertion of CVCs for a longer duration and thus act as risk factors for nosocomial infections. However, a higher incidence has been reported by authors such as Al-Gethamy et al. [22], who reported 24.06 CLABSI incidence, and Patil et al. [23], who reported 47.6.

The precise pattern of causative organisms varies across countries and between ICUs [24]. We found a preponderance of Gram‐negative bacteremia (77%) (Table 1), which was similar to the findings reported by Dasgupta et al. [25], who found 75%, and Kallel et al. [26], who found 89.6% predominance of Gram-negative organisms. However, in contrast to our study, authors such as Singh et al. [18] and Patil et al. [23] have reported Gram-positive bacteremia commonly associated with nosocomial BSI. The most common organisms isolated from CLABSI cases were *Acinetobacter *species (23%), followed by *Escherichia coli* (16.5%) and *S. aureus* (methicillin-resistant *Staphylococcus aureus* {MRSA}) (15.6%) (Table 2), and these findings were comparable to the studies reported by Mathur et al. [27] and Sahu et al. [28], who reported that *Acinetobacter* species commonly isolated organisms in CLABSI patients. The reason for the higher rate of *Acinetobacter *species associated with nosocomial bacteremia in the present study might be that the risk factors for *Acinetobacter *species infection are colonization; prolonged ICU stay; invasive procedures, commonly intravascular catheters; and the use of broad-spectrum antimicrobials. We observed a male predominance of 82.4% in patients with CLABSI as compared to females (17.6%). Similar findings were reported by Khurana et al., with 82.5% male predominance [29].

The risk factors for CLABSI are prolonged hospitalization, invasive procedures, prolonged duration of invasive devices, advanced age, comorbidities, and immunocompromised state. The common age group affected was 21-30 years, followed by 31-40 years, with a mean of 36.2±14.1 years (Table 3). Comparable finding was reported by Sahu et al., who found a mean age of 20.0±25.43 (from one month to 84 years) in CLABSI-infected patients [28]. There was no statistical significance in the age of the patients as compared to patients without nosocomial infections (Table 6), and similar findings were reported by Dasgupta et al. [25]. As limited information is available in the medical literature on nosocomial infections in trauma patients admitted to ICUs, in the present study, we observed that the young age group was commonly affected, which might be due to in the trauma ICU; mostly, young accidental patients with traumatic injuries were admitted who were on multiple invasive devices for longer duration. Some underwent surgeries, which predispose the patient to secondary bacteremia. Thus, all these factors might have rendered these patients prone to CLABSI.

Patients with longer length of ICU stay have a higher risk of developing nosocomial BSI. We observed the highest CLABSI cases from patients with longer length of ICU stay. The highest CLABSI cases were observed from patients with >11 days of ICU stay (77% of patients) with a mean of 19±18 days (Table 4). These results were comparable with the findings of Choudhuri et al., who reported that 70.11% (a mean of 15.24±10.9 days) of patients with ≥7 days of ICU stay developed nosocomial BSI [30]. Patients with prolonged central venous catheterization are at higher risk of acquiring nosocomial BSI. We observed the highest CLABSI cases in patients with a prolonged duration of central venous catheter of >11 days (56.8% of patients) with a mean of 18.13±10.02 days (Table 5). Our results were comparable with the findings of Rode et al., who reported nosocomial BSI in 77.58% of patients with >12 days of central venous catheter as compared to 22.42 patients with <12 days of central venous catheter [31]. The risk factors such as longer length of ICU stay and prolonged duration of central venous catheterization were statistically significant (Table 6). Our findings were similar with the studies of Dasgupta et al. [25] and Mishra et al. [32], who found that the risk factors such as longer length of ICU stay and prolonged duration of CVC, respectively, were statistically significant.

Patients with chronic illness have a greater chance of developing CLABSI. We observed that 58.1% of patients who developed CLABSI had no associated comorbidity, whereas only 49.1% of CLABSI cases had associated comorbidities. The most common comorbidity associated with CLABSI was diabetes mellitus (20.3%), followed by hypertension (14.8%) (Table 7). These findings were comparable with the study by Mishra et al. [32] and Singh et al. [33], who reported that diabetes mellitus (41% and 47.1%, respectively) and hypertension (39% and 32.7%, respectively) were associated with CLABSI. In our study, the greater frequency of CLABSI in diabetic patients might be due to uncontrolled sugar that leads to immune dysfunction and thus makes the patients more prone to nosocomial infections. Of the patients with CLABSI, 58.1% had no comorbidity in the TICU. The higher incidence rate of CLABSI in these trauma patients might be because in the trauma ICU, mostly, young (21-30 years) patients were admitted with traumatic injuries and were on multiple invasive devices for longer duration (mostly intravascular catheters) along with multiple surgeries. All these risk factors might have predisposed these patients to nosocomial infections (CLABSI).

Emerging multidrug-resistant organisms are another major challenge in the treatment of these infections. MRSA is highly prevalent in ICUs especially in postsurgical and trauma patient and are associated with poor outcome. We observed a 100% prevalence of MRSA, which was similar to the findings of Tomar et al., who reported 100% MRSA [34]. In our study, the reason for the high prevalence of MRSA might be that patients in the trauma ICU had wounds, drains, and invasive monitoring devices that cause breaches in the skin, which increases the risk of developing infections. We found that one (5.9%) isolate of *Staphylococcus aureus* was resistant to linezolid and one (14.3%) isolate of the *Enterococcus* species was resistant to teicoplanin. Out of the seven isolates of the *Enterococcus* species, two (26.6%) isolates were vancomycin-resistant (VRE). Comparable findings were reported by Deshpande et al. (19.6% VRE) [35]. The risk factors for VRE bloodstream infections include prolonged invasive devices particularly intravascular catheters, immunosuppression, and the overuse of antibiotics. Central line-associated bloodstream infections are associated with increased morbidity and mortality. We observed that the mortality rate of patients with CLABSI was 41.9% as compared to patients without nosocomial infections (63%). Some authors such as Dasgupta et al. [25] and Mishra et al. [32] have reported increased mortality associated with nosocomial infections, while others such as Rello et al. [36] have not shown higher mortality in patients with nosocomial infections. No statistical significance was observed in mortality rate in the patients with compared to those without nosocomial infection (Table 6), and similar findings were reported by Dasgupta et al. [25].

Our study emphasized on the healthcare-associated risk factors associated with CLABSI and incidence and pathogens causing central line-associated infections in trauma patients admitted to the trauma ICU. The limitation of the study was that bundle care could not be implemented in the study due to resource constrain.

## Conclusions

In our study, healthcare-associated risk factors such as longer length of ICU stay and prolonged duration of central venous catheterization were independent risk factors for developing central line-associated bloodstream infections. Apart from this, multidrug resistance to routine drugs was the highest, leaving with few treatment options. Therefore, the prevention of these nosocomial infections is required, which can be achieved not only by following preventive measures but also through surveillance programs that would help by detecting early nosocomial infections and the necessary actions required.
